# The bodily self in fibromyalgia: A systematic review and meta‐analysis of body image, body representation, and interoceptive dysfunction

**DOI:** 10.1111/pcn.70099

**Published:** 2026-07-02

**Authors:** Martina Mesce, Matteo Scalzeri, Carmen M. Galvez‐Sánchez, Julio A. Camacho‐Ruiz, Rosa M. Limiñana‐Gras, Federica Galli

**Affiliations:** ^1^ Department of Dynamic Clinical and Health Psychology, Sapienza University of Rome Rome Italy; ^2^ Department of Personality Evaluation and Psychological Treatment, Faculty of Psychology and Speech Therapy, University of Murcia Murcia Spain

**Keywords:** body image, fibromyalgia, interoception, meta‐analysis, systematic review

## Abstract

Fibromyalgia is a complex chronic pain condition associated with widespread symptoms and perceptual distortions beyond nociception. Alterations in body image, body representation, and interoception may contribute to symptom severity and reduced quality of life. This review synthesizes evidence related to body image in adults with fibromyalgia. A systematic review and meta‐analysis were conducted following PRISMA 2020 guidelines. PubMed, Scopus, and Web of Science were searched. Eligible studies included quantitative and qualitative designs examining body image, body awareness, and body esteem in adults with fibromyalgia. Risk of bias was assessed using the Newcastle‐Ottawa Scale and the CASP checklist. A random‐effects meta‐analysis was performed. Meta‐regression and subgroup analyses explored sources of variance. Twenty‐five studies were included. Patients with fibromyalgia showed significant impairments in body image, body awareness, and body esteem compared to healthy controls and other rheumatic conditions (pooled ES = 0.99; 95% CI [0.64–1.35]). These disturbances were positively associated with pain intensity and disease impact, and negatively with quality of life, anxiety, depression, and alexithymia. Qualitative findings described bodily alienation and transformation of bodily identity. Evidence was limited by cross‐sectional designs and variability in outcome measures. Fibromyalgia involves multidimensional disturbances of the bodily self with perceptual, cognitive, and affective components, associated with greater symptom burden and poorer quality of life. Longitudinal and mixed‐methods research integrating neurobiological and subjective measures is needed, alongside standardized tools and targeted interventions for body awareness and interoceptive regulation.

Fibromyalgia (FM) is one of the most complex and debilitating chronic pain conditions, characterized by widespread musculoskeletal pain, fatigue, sleep disturbances, mood disturbances (i.e., anxiety, depression, etc.), and cognitive dysfunction.^1^, ^2^, ^3^ Beyond these symptoms, patients with FM may experience alterations at a perceptual and representational level, including distorted body perception and reduced awareness of internal bodily state.^4^, ^5^ These alterations refer specifically to body‐related perceptual and cognitive processes and should not be conflated with somatic symptoms such as pain, fatigue, or sleep disturbances. These phenomena – including alteration in body image, body schema, and interoceptive functioning – have received increasing attention in recent years, suggesting a broader impairment of bodily self in patients with FM. Understanding these alterations in these patients is crucial because they directly influence pain perception, daily functioning, and quality of life^6^, ^7^, ^8^, ^9^ and may reduce the effectiveness of coping strategies, and interfere with pharmacological or rehabilitative treatment.^10^, ^11^, ^12^ Systematic mapping of these aspects can therefore guide personalized interventions and improve clinical outcomes.^13^, ^14^

The construct of body image has been defined by Schilder and Wertman^15^ as ‘the figuration of the body in the mind’, a dynamic representation continuously reconstructed through bodily and psychic experiences. Subsequent authors have emphasized that body image lies at the interface between representation and experience, encompassing both objective and subjective, conscious and unconscious elements.^16^, ^17^ A closely related evaluative dimension is body esteem, which refers to the affective and evaluative attitudes toward one's own body. In line with Schilder and Wertman,^15^ who argued that physical pain can alter body image, FM has often been associated with alterations in body perception and body‐related evaluative processes, including low body esteem and negative attitudes toward the self, rather than somatic symptom changes *per se*.^6^, ^7^

The literature has noted that FM patients suffer from body perception disorders, such as the sensation of phantom swelling in the hands or feet, despite the absence of observable changes.^8^, ^9^, ^18^ Such experiences suggest a disconnect between somatic signals and the visual representation of the body.

The bodily self is inherently multidimensional construct, encompassing perceptual, cognitive, and emotional components,^19^, ^20^, ^21^ which may be altered in FM, particularly in relation to painful or stiff areas of the body.^22^ It is typically described as comprising two main representational components: body image (perceptual‐emotional representation) and body schema (implicit sensory‐motor representation that guides action).^23^, ^24^, ^25^ These two components are conceptualized here as distinct but interacting dimensions within the broader construct of body representation. The body schema involves the integration of visual, tactile, proprioceptive, vestibular, and motor signals^26^; distortions of this system have been documented in other chronic pain conditions (e.g., back pain, region‐specific pain), where they contribute to pain maintenance and functional impairment.^27^, ^28^, ^29^ In this review, the term ‘body awareness’ is not used as a synonym of body schema, but as an umbrella term referring to conscious access to bodily signals, including both interoceptive and exteroceptive components, depending on the operationalization of the instruments used in the included studies.

A further central element of the bodily self is interoception, defined as the processing of internal bodily signals by the central nervous system, encompassing both conscious and unconscious levels.^30^, ^31^ Building on the taxonomy proposed by Garfinkel and colleagues,^32^ interoception comprises at least three distinguishable components: interoceptive accuracy (objective performance on interoceptive tasks, typically measured behaviorally); interoceptive sensibility (the subjective tendency to attend to and handle bodily signals, assessed through self‐report); and interoceptive awareness (metacognitive insight into the correspondence between one's accuracy and sensibility).^32^, ^33^, ^34^ Interoceptive dysfunction has been related to several clinical conditions, including depression, eating disorders, somatoform disorders, and hypochondria.^35^, ^36^, ^37^ Although traditionally studied in relation to emotion regulation and cognition, recent evidence suggests that interoception also influences body image, body schema, and embodiment.^10^, ^38^

In chronic pain disorders (e.g., chronic musculoskeletal pain, central pain syndrome, and FM), interoception is often impaired: patients show dysfunctional internal awareness, characterized by hypervigilance to bodily sensations or poor interoceptive accuracy, which contributes to pain amplification and distorted body perception.^11^, ^12^

Despite growing interest in these aspects, to our knowledge, there is currently no systematic synthesis of the literature on bodily self in patients with FM. Filling this research gap is clinically relevant, as alterations in body image, body schema, and interoceptive awareness are increasingly recognized as key mechanisms contributing to the maintenance and exacerbation of symptoms in patients with FM. A comprehensive synthesis of the available evidence can therefore provide clinicians and researchers with a clearer understanding of how alterations in bodily self interact with pain perception, emotional regulation, and functional outcomes in FM. The objective of this systematic review and meta‐analysis is to synthesize and quantitatively and qualitatively evaluate the available evidence on body image and body esteem, body schema and perceptual body representation, and body awareness and interoceptive functioning in adults with FM, as well as to examine their associations with clinical and psychological outcomes. The findings of this review may also inform the development of targeted psychological and rehabilitative interventions addressing disturbances in bodily self‐experience within a multimodal treatment framework for FM.

## Methods

### Search Strategy

From May to July 2025, a systematic literature review was conducted by searching the PubMed, Scopus, and Web of Science databases; an updated search was performed on April 23, 2026, to capture any newly published studies. To ensure methodological rigor and accuracy, this review followed the PRISMA guidelines.^39^ The PRISMA checklist is provided in Supporting Information.

The search keywords included ‘fibromyalgia’, ‘self‐awareness’, ‘self‐perception’, ‘body image’, ‘body perception’, ‘body representation’, ‘self‐concept’, and ‘interoception’, combined with the Boolean operators ‘AND’ and ‘OR’. The review protocol was registered in the PROSPERO database (registration ID: CRD420251105050).

Deviations from the pre‐registered protocol were documented and reported below. Specifically, although pre‐registration indicated the exclusion of studies primarily focused on interoception, this criterion was revised during the review process to include studies assessing subjective interoceptive experience (e.g., interoceptive sensibility), as they are conceptually aligned with the broader construct of the bodily self. Conversely, studies assessing exclusively objective or performance‐based interoceptive accuracy without reference to subjective experience were excluded. Furthermore, studies solely aimed at validating psychometric instruments without providing group comparisons or usable outcome data were not included in the meta‐analysis, although they were considered in the qualitative synthesis where relevant.

### Eligibility Criteria

Given the observational nature of the included studies, a PEO (Population‐Exposure‐Outcome) framework was adapted to structure the research question. This choice was considered more appropriate than the PICO framework, as the present review did not aim to evaluate interventions but rather to synthesize observational evidence on bodily‐self dimensions in FM

- Population (P): studies involving adult participants (≥18 years) diagnosed with FM (without distinction of sex).
- Exposure (E): FM as a chronic pain condition characterized by persistent widespread pain and associated symptomatology (e.g., fatigue, sleep disturbances, and cognitive symptoms). In comparative studies, exposure was operationalized as the presence of FM relative to healthy controls or individuals with other chronic pain conditions.
- Outcomes (O): bodily‐self dimensions in patients with FM, operationalized into three domains: (i) body image and body esteem; (ii) body schema and perceptual body representation; and (iii) body awareness/interoception, assessed through subjective or self‐report measures where applicable and through performance‐based measures for body schema‐related constructs.

Furthermore, the search was not limited to a specific time: it included all published studies.

Eligibility criteria included peer‐reviewed publications reporting different experimental designs, including quantitative (observational, cross‐sectional, correlational), qualitative, and mixed‐method studies, provided they provided data on the subjective bodily experience of patients with FM. The following study designs were considered grounds for exclusion: case reports, conference abstracts, editorials, opinion pieces, and reviews; duplicate studies; studies not focused on FM or that included mixed samples without distinguishing FM‐related data; studies focusing on non‐bodily constructs of the self (such as self‐efficacy, self‐esteem) that were not explicitly related to body perception or bodily self; studies focusing exclusively on objective, performance‐based, or neurophysiological indicators of interoception or body schema (e.g., tactile or proprioceptive tasks) without any link to subjective bodily experience; clinical interventions (i.e., pharmacological, rehabilitative, or psychotherapeutic) that did not measure or report data related to bodily experience or bodily self; studies on children or adolescents. In line with established review methodologies,^40^ studies published in languages other than English were excluded from the review.

### Data Extraction and Quality Assessment

All records were independently screened by two reviewers (M.M. and C.M.‐G‐S.). In the initial phase, titles and abstracts were reviewed to exclude studies that did not meet the inclusion criteria. Subsequently, the full texts of potentially eligible articles were evaluated in detail according to predefined criteria. Any disagreements were resolved through discussion until consensus was reached.

Data extraction was performed independently by the reviewers themselves. The data extracted included: author, year of publication, methodological design, sample size, participant characteristics (as defined by the PEO framework), body image constructs (body image, body schema and perceptual body representation, body awareness, and interoception), and assessment tools used. Outcomes were categorized into three main domains: (i) body image and body esteem; (ii) body schema and perceptual body representation; and (iii) body awareness/interoception (e.g., interoceptive sensibility, awareness of internal bodily signals). Only outcomes that could be meaningfully assigned to these categories and measured using validated self‐report instruments were considered for quantitative synthesis; for the meta‐analysis specifically, only outcomes derived from validated self‐report instruments were included, ensuring conceptual and methodological comparability across studies. When multiple outcomes for the same construct were reported within a single study, all relevant effect sizes were retained and modeled using a multilevel approach, avoiding arbitrary selection or aggregation.

Any inconsistencies in the extracted data were resolved through discussion and consensus; no automated tools were used. It was not necessary to contact the study authors, as all the required information was available. No missing or unclear data were found, so data imputation was not necessary.

The risk of bias in the studies included was assessed using different tools depending on the study design, in particular: for cross‐sectional studies, the JBI Critical Appraisal Checklist for Analytical Cross‐Sectional Studies,^41^ for RCTs, the Cochrane Risk of Bias 2,^42^ for qualitative studies, the JBI Critical Appraisal Checklist for Qualitative Research,^43^ and for the only mixed‐methods study included, the Mixed Methods Appraisal Tool.^44^

### Data synthesis

Quantitative data were summarized using descriptive statistics (means and standard deviations), where available, to describe sample characteristics and provide an exploratory comparison of the levels of relevant variables.

We performed a meta‐analysis to estimate the magnitude of differences (expressed as Cohen's *d*, with positive values indicating higher scores in FM patients relative to controls) in bodily‐self dimensions between patients with FM and control groups. In order to ensure greater methodological consistency and comparability of the data, studies were included in the meta‐analysis only if they: (i) provided sufficient quantitative data to compute effect sizes; and (ii) included a healthy control comparison group; studies that did not provide a direct comparison between FM patients and a control group (e.g., validation studies without group comparisons) were included in the systematic review but excluded from the meta‐analysis.

Following this assessment, we identified eight studies eligible for meta‐analysis, covering two domains: body awareness/interoception (*k* = 6) and body image/body esteem (*k* = 2). We grouped together works investigating interoception and body awareness, as body awareness theoretically encompasses interoceptive dimensions (see Table 1). Although conceptually distinct, these constructs were grouped for meta‐analytic purposes due to overlapping in their self‐report operationalizations. For each construct, only outcomes derived from validated self‐report measures were included in the quantitative synthesis, ensuring conceptual and methodological comparability across studies.

**Table 1:** Operational definitions of bodily self‐related constructs included in the review

Concept	Definition
Body awareness	Conscious access to and attention toward bodily signals, including both interoceptive and exteroceptive information
Body esteem	Affective and evaluative dimension of body image reflecting attitudes toward one's own body
Body image	Dynamic perceptual and emotional representation of the body, encompassing subjective experience, beliefs, and attitudes toward one's body
Body representation	Umbrella construct referring to the multidimensional representation of the body, including perceptual, cognitive, and affective components
Body schema	Implicit, sensorimotor representation of the body that supports action and spatial orientation through integration of multisensory inputs
Interoception	Processing of internal bodily signals (e.g., cardiac, respiratory) by the central nervous system, at both conscious and unconscious levels

Body schema‐related constructs were excluded from the meta‐analysis due to their substantial methodological heterogeneity, their performance‐based nature, and their limited comparability with subjective self‐report measures of bodily experience. Additionally, available data was insufficient and inconsistently reported, preventing reliable computation and aggregation of effect sizes. Findings from body schema studies were nonetheless synthesized narratively in the qualitative synthesis.

Since three studies^45^, ^46^, ^47^ provided multiple effect sizes, we employed a multilevel mixed‐effects model in which effect sizes were nested within studies; this allowed us to include multiple outcomes per study without aggregating or selectively choosing a single effect size. In addition, construct type was included as a fixed moderator to account for conceptual differences across domains.

The analyses were performed using the JASP Meta‐Analysis module (version 0.19.3). We assessed heterogeneity among studies using Cochran's *Q* test. In addition, the *I* ^2^ statistic was calculated to quantify the proportion of total variance attributable to between‐study heterogeneity. We then estimated the pooled effect size of the considered studies using the Restricted Maximum Likelihood (REML) method. To assess the influence of individual studies on the overall results, a leave‐one‐out sensitivity analysis was conducted by iteratively removing one study at a time and recalculating the pooled effect size. This analysis did not reveal substantial changes in heterogeneity or in the statistical significance of the pooled estimates, suggesting that no single study exerted undue influence on the overall results.

The possible presence of publication biases was assessed through Egger's test and a funnel plot to visually check for symmetry.

## Results

To provide an integrated view of empirical evidence, findings are presented according to the main dimensions of the bodily self‐explored across studies, encompassing body image and esteem, body schema and perceptual aspects of body representation, and body awareness and interoceptive processes. Quantitative and qualitative results are reported separately, followed by an evaluation of study quality and a meta‐analytic review of the findings.

### Literature Search

A total of 6727 records were identified. After removing duplicates, titles and abstracts were screened against the inclusion criteria. The full texts of 42 potentially eligible articles were retrieved. Studies were excluded for the following reasons: (a) they investigated constructs not directly relevant to the present review; (b) they did not use standardized measures; (c) the data were reported in aggregate form and specific results for the FM group were unavailable or insufficient; (d) the article was a validation study of a measurement instrument; or (e) the study reported the results of a psychological intervention and therefore did not fall within the scope of this review. Therefore, 25 articles^18^, ^22^, ^45^, ^46^, ^47^, ^48^, ^49^, ^50^, ^51^, ^52^, ^53^, ^54^, ^55^, ^56^, ^57^, ^58^, ^59^, ^60^, ^61^, ^62^, ^63^, ^64^, ^65^, ^66^, ^67^ were included, published between 2003 and 2025. The selection procedure is summarized in the PRISMA flow diagram (Fig. 1).

**Fig. 1:**
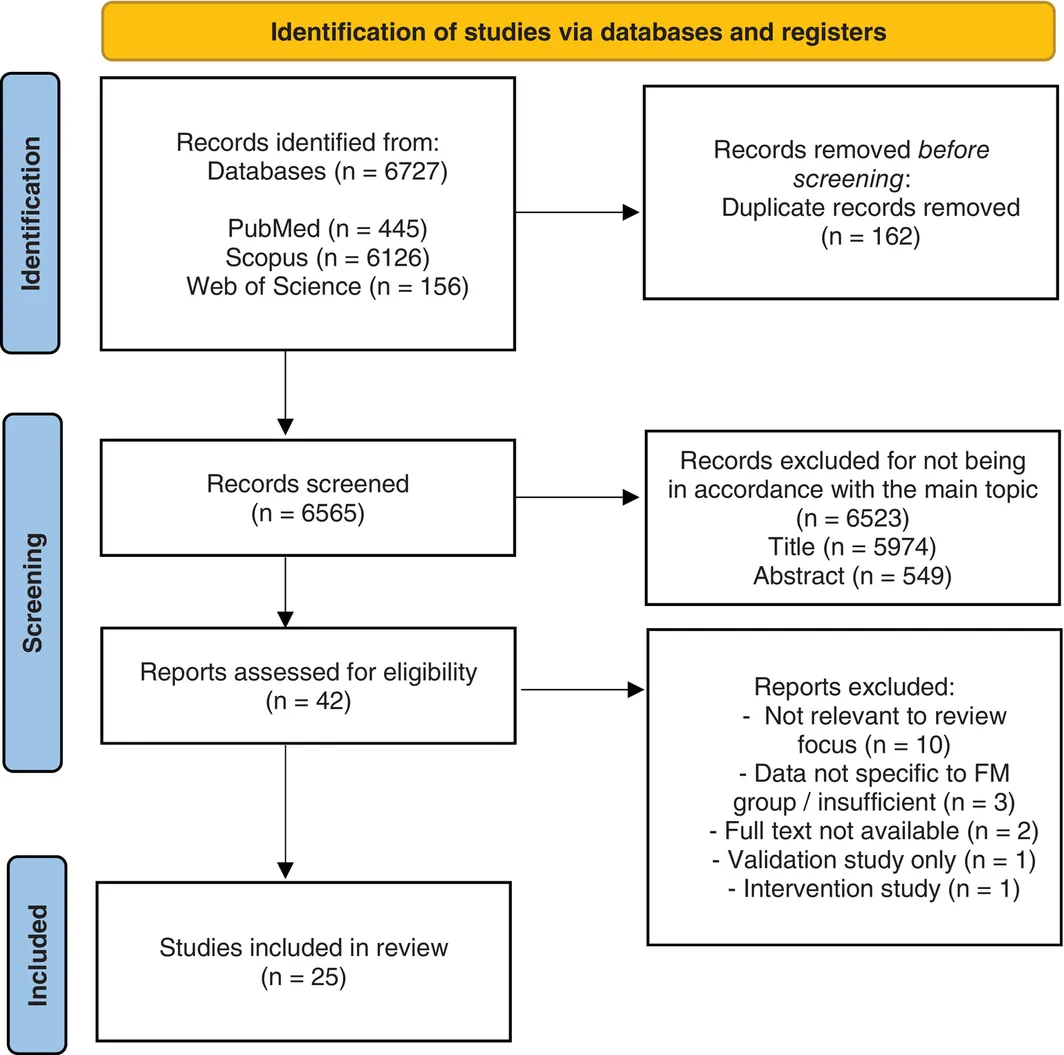
Flowchart.

The main characteristics of the included studies are summarized in Table 2. This review included both quantitative and qualitative studies, and the results are presented below. Taken together, the quantitative and qualitative results present a complex picture of bodily self alteration in FM, highlighting widespread alterations involving body image, body schema, and perceptual body representation, and interoceptive awareness.

**Table 2:** Summary of included studies

Study Author. Year	Design	Sample (*N*; gender*; age)	Instruments	Construct	Other variables	Main results
Agostinho *et al*., 2025	Cross‐sectional	FM = 29 50.41 ± 10.34	Hearbeat detection task; *ad hoc* questions; MAIA	Body awareness/interoception	Cognitive assessment, pain, FM impact, depression, anxiety	Higher interoceptive accuracy related to better cognitive performance, while greater interoceptive sensibility/awareness related to poorer cognition, moderated by symptom severity.
Akkaya *et al*., 2012	Cross‐sectional	FM = 51 35.5 ± 9.9 CG = 41 33.3 ± 7.4	BIS	Body image and body esteem	Pain, FM impact, depression, quality of life	Body image disturbance was significantly associated with higher pain intensity, greater functional impairment, more severe depressive symptoms, and lower quality of life in FM patients.
Augière *et al*., 2024	Cross‐sectional	FM = 20 19 females 44.0 CG = 24 23 females 42.5	TDE tasks; body drawings	Body schema and perceptual body representation	Physical activity, pain	Despite distortions in body representation, patients with FM showed preserved tactile distance estimation, suggesting partial dissociation between body representation and tactile spatial processing.
Berk *et al*., 2020	Cross‐sectional	FM = 141 42.18 ± 4.6 CG = 30 41.93 ± 5.5	BIQ	Body image and body esteem	FM impact, anxiety, depression, quality of life	Greater disease severity correlated with poorer body perception, increased depressive and anxiety symptoms, and reduced quality of life, indicating that negative body evaluation may be linked to illness burden.
Borg *et al*., 2015	Cross‐sectional	FM = 18 50.4 ± 9.9 CG =18 49.5 ± 7.8	MAIA; SSAS; SCS	Body awareness/interoception	Cognitive assessment, anxiety, depression, pain, catastrophizing	Patients exhibited increased attentional focus on internal sensations and subjective interoceptive experiences, indicating interoceptive hypervigilance despite ambiguous bodily cues.
Borg *et al*., 2018	Cross‐sectional	FM = 21 50.3 ± 8.7 CG = 21 46.3 ± 9.9	Mental heartbeat tracking task; MAIA; SSAS; SCS	Body awareness/interoception	Heart rate at rest, anxiety, depression, emotional reaction, pain, auditory selective attention task	Lower interoceptive accuracy was predicted by higher pain intensity and emotional distress, highlighting the role of pain and emotion in interoceptive deficits in FM.
Boyington *et al*., 2015	Comparative qualitative	27 (in particular: 11 FM, 10 RA, 6 FM + RA) 55.9 ± 9.1	Semi‐structured interview	Body image and body esteem (qualitative)	Pain	Women with FM reported more negative body image perceptions than those with rheumatoid arthritis, with racial differences indicating sociocultural influences on body image appraisal.
Calsius *et al*., 2015	Phenomenological qualitative	FM = 15 (9 with alexithymia)	IPA	Body awareness/interoception (qualitative)	Alexithymia, anxiety, depression	Qualitative data revealed that patients with alexithymia experience their bodies as fragmented, alien, and difficult to sense, reflecting a disrupted embodied self‐awareness.
Duschek *et al*., 2017	Cross‐sectional	FM = 45 49.93 ± 8.81 CG = 31 47.13 ± 9.38	Heartbeat detection task	Body awareness/interoception	FM impact, SCID, attentional and executive function	Patients with FM exhibited reduced interoceptive awareness compared to controls, which was related to autonomic dysregulation and emotional symptoms.
Horwitz *et al*., 2003	Hermeneutic phenomenological	FM = 8 46 ± 5.6	Video self‐interpretation; semi‐structured interview	Body image and body esteem (qualitative)	/	Video self‐interpretation elicited ambivalent reactions: patients perceived their bodies as strange or unfamiliar, yet the process also facilitated new self‐understanding and emotional integration.
Kircali *et al*., 2024	Randomized Controlled Trial	FM = 50	BAQ	Body awareness/interoception	Pain, kinesiophobia, anxiety, depression, cognitive‐mood, self‐esteem	Pain neuroscience education combined with motor imagery exercises improved body perception and functional outcomes, suggesting that body‐focused cognitive‐motor interventions can modulate pain and embodiment.
Martínez *et al*., 2018	Cross‐sectional	FM = 14 54.35 ± 1.89 CG = 13 53.86 ± 3.30	RHI; BES; BPQ	Body schema and perceptual body representation; body image and body esteem	Pain, FM impact, quality of life	Patients showed disturbed somatorepresentations and increased body schema plasticity, supporting the concept of an “embodied pain” where altered bodily representations contribute to chronic pain.
Martínez *et al*., 2019	Cross‐sectional	FM = 14 50.14 ± 12.47 CG = 14 51.64 ± 7.02	Left/Right Judgment Test; TPD test; Mirror Test	Body schema and perceptual body representation	Pain, FM impact, quality of life	Participants demonstrated a distorted body schema and a higher tendency to experience anomalous somatosensory sensations, pointing to instability in body representation mechanisms.
Martínez *et al*., 2022	Randomized Controlled Trial	FM (tactile discrimination) = 13 44.31 ± 9.42 CG (FM assigned to tactile stimulation) = 13 50.69 ± 7.69	BES; BPQ	Body image and body esteem	Pain, FM impact	Short‐term modulation of the body schema through sensorimotor training led to reductions in pain intensity, indicating that improving body representation can alleviate symptoms.
Menten *et al*., 2022	Randomized Controlled Trial	FM = 24 (13 aerobic group; 11 pilates group) 48.3 ± 8.4 CG = 24 40.1 ± 10.9	Drawing Test; TPD	Body schema and perceptual body representation	Pain	Contrary to expectations, patients did not show consistent distortions in body image or tactile acuity, suggesting heterogeneity in perceptual alterations among FM individuals.
Rost *et al*., 2017	Cross‐sectional	FM = 47 (39 females) 45.5 ± 9.2 CG = 45 (37 females) 44.9 ± 12.2	BVS; Heartbeat tracking task	Body awareness/interoception	Pain, depression, anxiety and stress, catastrophic thinking, HRV	Although interoceptive accuracy was normal, patients displayed reduced self‐regulatory capacity and generalized hypervigilance, indicating dissociation between bodily accuracy and regulation.
Scandola *et al*., 2022	Cross‐sectional	FM = 30 48.45 ± 10.92 CG = 30 49.9 ± 10.22	BoFI; VMIQ	Body schema and perceptual body representation	Pain, FM impact, anxiety, depression	FM patients experienced stronger bodily illusions and altered motor imagery performance, consistent with disrupted multisensory integration and body ownership.
Schmitz *et al*., 2021	Cross‐sectional	FM = 55 CG = 55	MAIA	Body awareness/interoception	Depression, anxiety, alexithymia, emotional regulation	Interoceptive sensibility was lower in FM and correlated with higher alexithymia and poorer emotion regulation, suggesting impaired emotional–bodily integration.
Świdrak *et al*., 2023	Cross‐sectional	FM = 21 43 ± 5	MAIA; FBAQ; VR paradigm	Body awareness/interoception; body schema and perceptual body representation	FM impact, pain	Virtual embodiment significantly reduced pain and improved body ownership, showing that immersive body illusions can temporarily normalize body representation.
Świdrak *et al*., 2025	Mixed‐methods	FM = 28 48 ± 13	BES; MAIA; FBAQ; BDES	Body image and body esteem; body schema and perceptual body representation; body awareness/interoception	FM impact, pain	Mapping tasks revealed distinct patient subtypes with specific distortions in body image and body schema; emotional valence of the drawings correlated with pain and affective symptoms.
Todd *et al*., 2024	Cross‐sectional	FM = 154 (144 females) 42.13 ± 12.00 CG = 94 40.09 ± 8.21	PAT	Body awareness/interoception	FM severity and impact, task‐related item	Contrary to prior reports, individuals with FM showed heightened interoceptive accuracy, possibly reflecting interoceptive hypervigilance rather than awareness deficits.
Valenzuela‐Moguillansky, 2013	Phenomenological qualitative	FM = 12 11 females 55.4 ± 10.7	Elicitation interview	Body awareness/interoception (qualitative)	/	Patients described an alienated and fragmented bodily experience, marked by disconnection from internal sensations and difficulty inhabiting the body.
Valenzuela‐Moguillansky *et al*., 2017	Cross‐sectional	FM = 30 46.77 ± 12.66 CG = 29 43.52 ± 10.97	Body‐Scaled Action Task; Heartbeat detection task; MAIA	Body schema and perceptual body representation; body awareness/interoception	FM impact, pain, depression, anxiety	FM was associated with reduced interoceptive and exteroceptive body‐self awareness, suggesting a global disruption of embodied self‐experience.
Van Overmeire *et al*., 2022	Cross‐sectional	FM = 103	BIS	Body image and body esteem	Mental health, FM impact	Women with FM reported significantly poorer body image and higher medication use, which were associated with worse mental health indicators.
Viceconti *et al*., 2022	Cross‐sectional	FM = 854 (803 females) 48.1 ± 10.5	*Ad‐hoc* questionnaire	Body schema and perceptual body representation	FIQ; Neuropathic Pain Symptoms	Self‐reported body perception distortions correlated positively with neuropathic pain features, supporting a link between altered body representation and central sensitization.

When gender is not specified, the sample consists of women. The “Construct” column reflects the categorization adopted in the present review, which may differ from labels used by original authors. The MAIA was classified as a measure of interoceptive sensibility across all studies; heartbeat detection/tracking tasks and the PAT as measures of interoceptive accuracy, in line with Garfinkel *et al*. (2015); qualitative studies were assigned to the domain most closely reflected by their thematic content.

BAQ, Body Awareness Questionnaire; BDES, Body Drawing Evaluation Scale; BES, Body Esteem Scale; BIQ, body image questionnaire; BIS, Body Image Scale; BPQ, Body Perception Questionnaire; BoFI, Body Feelings and Illusions questionnaire; BVS, Body Vigilance Scale; CG, Control Group; FBAQ, Fremantle Back Awareness Questionnaire; FM, fibromyalgia; IPA, Interpretative Phenomenological Analysis; MAIA, Multidimensional Assessment of Interoceptive Awareness; PAT, Phase Adjustment Task; RA, rheumatoid arthritis; RHI, Rubber Hand Illusion; SCID, Structured Clinical Interview for the DSM; SCS, Self‐Consciousness Scale; SSAS, Somatosensory Amplification Scale; TDE, tactile distance estimation; TPD, two‐point discrimination; VMIQ, Visual Motor Imagery Questionnaire.

### Quantitative evidence

Nineteen quantitative studies^18^, ^22^, ^45^, ^46^, ^47^, ^48^, ^49^, ^50^, ^51^, ^54^, ^56^, ^57^, ^58^, ^59^, ^60^, ^61^, ^62^, ^64^, ^66^, ^67^ and the quantitative component of a mixed study^63^ were included. Despite the use of different tools and the inclusion of heterogeneous samples, these studies allow us to identify recurring patterns in multiple dimensions of embodied experience in patients with FM. The distribution of studies across constructs was as follows: body image and body esteem (*k* = 7), body schema and perceptual body representation (*k* = 9), and body awareness/interoception (*k* = 11), with some studies addressing multiple domains simultaneously. Table 2 also provides an overview of the operationalizations used for each target construct, distinguishing between self‐report and performance‐based measures.

In the following sections, the results are structured according to the main theoretical areas examined: body image and body esteem, body schema and perceptual body representation, and body awareness and interoception.

#### Body Image and Body Esteem

According to the studies included patients with FM showed systematic impairments in body image and body esteem compared to healthy controls. Body image disturbances were significantly more pronounced in patients with FM and positively correlated with pain intensity and disease impact, and negatively correlated with quality of life and mental health indicators (e.g., anxiety, depression, alexithymia).^49^ Similar associations emerged with anxiety, depression, and overall health status,^45^, ^67^ with Van Overmeire and colleagues^67^ identifying mental health as the main predictor of body image disturbances.

Body esteem was consistently reported to be significantly lower in FM compared to healthy controls, with more pronounced deficits observed in the most painful body regions.^47^ In studies without control groups, body esteem showed negative correlations with negative emotions and body schema disturbances while demonstrating positive associations with interoceptive awareness; stronger correlations were observed in painful *versus* non‐painful body regions.^63^

All findings reported in this section derive exclusively from self‐report measures, ensuring consistency with the conceptual definition of body image and body esteem as subjective constructs.

#### Body Schema and Perceptual Body Representation

Body schema and perceptual body representation alterations in FM encompass both perceptual distortions and sensorimotor dysfunction, reflecting disruptions in how the body is mentally mapped and represented.^50^, ^61^, ^63^ The following section presents the main findings related to the aforementioned aspects.

Several studies documented subjective distortions in perceptual body representation among FM patients, including perceptions of phantom swelling, asymmetry, heaviness, lack of definition in body parts, and feelings of alienation or disownership.^50^, ^59^, ^61^, ^63^ These perceptual distortions were significantly more frequent in FM compared to healthy controls.^61^ Body drawing tasks revealed more representational alterations in FM participants compared to healthy controls, despite no significant group differences in tactile distance estimation tasks.^50^ Similarly, Menten and colleagues^59^ found no differences between FM and healthy controls in two‐point discrimination thresholds, although FM patients reported marked subjective changes in body perception, including feelings of distortion, altered size, and reduced clarity of body boundaries.

Additional phenomena, such as experiences of disownership, somatoparaphrenia‐like symptoms, and illusory sensations of movement, were described more frequently in FM patients, indicating alterations in the multisensory integration processes underlying perceptual body representation.^61^

FM patients demonstrated significant impairments as assessed through body schema tasks (i.e., Tactile Distance Estimation^50^; Left/Right Judgment Test^57^; Rubber Hand Illusion^47^; Body‐Scaled Action Task^66^; Two‐Point Discrimination Test.^57^, ^59^ Martínez and colleagues^57^ reported that FM patients performed significantly worse than pain‐free controls in right–left discrimination tasks, showing both reduced accuracy and prolonged response times. Additionally, FM patients exhibited elevated tactile thresholds and abnormal sensations (e.g., tingling or pain) when observing movement during motor imitation tasks, suggesting disruption of the functional and dynamic aspects of body schema.^57^ The Rubber Hand Illusion paradigm^47^ is reported here, as it primarily indexes multisensory integration and bodily ownership – processes belonging to body schema rather than interoceptive awareness. FM patients showed altered susceptibility to it compared to healthy controls, suggesting disruptions in the integration of tactile and visual signals underlying the sense of body ownership.

FM patients also showed difficulties in motor imagery and kinesthetic representation tasks (imagine performing actions from a third‐person perspective, as if you were observing yourself from an external position), with impairments correlating with symptom severity and disease duration, but not with anxiety or depression levels.^61^ Disturbances in body schema correlated negatively with body esteem and confidence, and positively with measures of disease severity and impact.^63^

These findings highlight a dissociation between objective performance‐based measures (often preserved or only mildly impaired) and subjective alterations in perceptual body representation, which appear to be more consistently disrupted in FM. All performance‐based tasks (e.g., tactile discrimination, proprioceptive judgments, motor imagery) are reported exclusively in this section to ensure conceptual consistency.

#### Body Awareness and Interoception

The following section presents the findings of studies that examine body awareness and interoceptive processes in FM. A substantial number of studies investigated body awareness and interoceptive processes in FM, yielding complex and sometimes paradoxical findings that varied according to assessment methodology.^18^, ^22^, ^46^, ^47^, ^51^, ^54^, ^56^, ^58^, ^60^, ^62^, ^63^, ^64^, ^66^

Studies using the Multidimensional Assessment of Interoceptive Awareness (MAIA^68^;) revealed a consistent pattern in FM patients characterized by: elevated scores on the ‘noticing’ subscale (indicating heightened attention to bodily sensations) but significantly lower scores on ‘trusting’ (confidence in body signals), ‘attention regulation’ (ability to sustain attention to body sensations), and ‘not distracting’ (tendency not to ignore body sensations for distraction) subscales compared to healthy controls.^46^, ^62^, ^66^ However, one study reported no significant differences in MAIA scores between FM and healthy control groups.^51^

These patterns of interoceptive awareness showed significant negative correlations with body esteem, vigilance to bodily signals, and various psychopathological measures, including emotional regulation, alexithymia, depression, and anxiety,^62^, ^63^, ^66^ suggesting interconnections between interoceptive dysfunction and broader aspects of embodied experience.

Experimental studies documented reduced body perception accuracy in FM compared to healthy controls, with performance positively correlated with pain intensity.^47^ Within‐FM designs revealed frequent perceptual distortions, including sensations of heaviness, swelling, and constriction,^18^ though intervention studies showed non‐significant pre‐post changes.^58^

FM patients demonstrated greater susceptibility to bodily illusions in virtual reality environments compared to healthy controls, suggesting altered multisensory integration processes underlying body awareness.^22^, ^47^, ^60^

Regarding objective measures of cardiac interoception, heartbeat detection tasks generally showed no significant differences in accuracy between FM patients and healthy controls,^46^, ^60^, ^66^ although some studies reported altered heart rate patterns in FM.^54^, ^60^ Notably, Todd and colleagues^64^ identified a dissociation pattern, with task‐based interoceptive performance linked to lower symptom impact, while interoceptive sensibility was associated with higher symptom severity. Objective measures of interoceptive accuracy (e.g., heartbeat detection tasks) are treated separately from subjective measures of interoceptive sensibility and awareness, in line with the taxonomy of Garfinkel and colleagues,^32^ as they reflect conceptually and operationally distinct constructs.

Additionally, a recent study linked interoceptive processes in FM to cognitive performance: higher interoceptive accuracy was associated with better short‐term memory and inhibition, whereas greater interoceptive sensibility and awareness were related to poorer working‐memory and inhibition scores, with accuracy‐cognition associations moderated by symptom severity.^48^

### Qualitative evidence

Four qualitative studies,^52^, ^53^, ^55^, ^65^ along with the qualitative component of a mixed‐methods study,^63^ were also included. Despite employing different methodological approaches – such as phenomenological, hermeneutic, and comparative – these investigations converge on recurring themes that delineate a complex and multidimensional understanding of the bodily self in individuals with FM. Subjective impact and overall dysfunction were also confirmed. FM patients often describe their bodies as ‘unreliable’, ‘treacherous’, or ‘foreign’, expressing dissatisfaction with their appearance and bodily functioning.^52^, ^53^, ^55^ Compared to patients with rheumatoid arthritis, those with FM report a more pronounced impact of symptoms on physical, emotional, and social levels, focusing on overall body function rather than aesthetic or localized aspects.^52^

Related to altered body awareness and body boundaries, it has been observed that in patients with FM, disturbances in body ownership, body awareness, and body boundaries are common, including experiences range from a vague body perception to a profound sense of alienation,^53^, ^65^ with descriptions of ‘disembodiment’, loss of control, and detachment from the body. Świdrak and colleagues^63^ identify a subgroup (called ‘Disconnected Body’) characterized by frequent body distortions and altered body representations in drawings.

Considering body alienation and emotional ambivalence, many individuals with FM report a ‘hyper‐presence’ of the painful body, experienced as a ‘broken machine’ or as something that no longer belongs to the self.^53^ In addition, ambivalent emotions toward the body – such as anger, frustration, or shame – are common in those suffering from FM, especially in relation to pain, fatigue, and changes in physical appearance.^55^

To conclude this section and regarding body identity and transformation in individuals with FM, chronic pain is experienced as a transformative factor, capable of redefining body identity over time. In fact, some patients report processes of resignation, redefinition, or reintegration of the bodily self.^55^, ^63^ Overall, qualitative findings converged with quantitative evidence in highlighting disturbances across all three dimensions examined – body image, body schema, and perceptual body representation, and body awareness – reinforcing the multidimensional nature of bodily‐self alterations in FM.

### Quality of Selected Studies and Risk of Bias

The risk of bias of the included studies was assessed using design‐specific tools (Table 3). Overall, the methodological quality of the literature was heterogeneous.

**Table 3:** Risk of bias assessment

Study	Design	Risk of bias tool	Overall risk of bias
Agostinho *et al*., 2025	Cross‐sectional	JBI Checklist – Cross‐sectional	Low risk
Akkaya *et al*., 2012	Cross‐sectional	JBI Checklist – Cross‐sectional	Moderate risk
Augière *et al*., 2024	Cross‐sectional	JBI Checklist – Cross‐sectional	Low risk
Berk *et al*., 2020	Cross‐sectional	JBI Checklist – Cross‐sectional	Moderate risk
Borg *et al*., 2015	Cross‐sectional	JBI Checklist – Cross‐sectional	Moderate risk
Borg *et al*., 2018	Cross‐sectional	JBI Checklist – Cross‐sectional	Low risk
Boyington *et al*., 2015	Comparative qualitative	JBI Checklist – Qualitative	High risk
Calsius *et al*., 2015	Phenomenological qualitative	JBI Checklist – Qualitative	Moderate risk
Duschek *et al*., 2017	Cross‐sectional	JBI Checklist – Cross‐sectional	Low risk
Horwitz *et al*., 2003	Hermeneutic phenomenological	JBI Checklist – Qualitative	Moderate risk
Kircali *et al*., 2024	Randomized Controlled Trial	Cochrane RoB 2	Low risk
Martínez *et al*., 2018	Cross‐sectional	JBI Checklist – Cross‐sectional	Moderate risk
Martínez *et al*., 2019	Cross‐sectional	JBI Checklist – Cross‐sectional	High risk
Martínez *et al*., 2022	Randomized Controlled Trial	Cochrane RoB 2	High risk
Menten *et al*., 2022	Randomized Controlled Trial	Cochrane RoB 2	Some concerns^†^
Rost *et al*., 2017	Cross‐sectional	JBI Checklist – Cross‐sectional	Moderate risk
Scandola *et al*., 2022	Cross‐sectional	JBI Checklist – Cross‐sectional	Moderate risk
Schmitz *et al*., 2021	Cross‐sectional	JBI Checklist – Cross‐sectional	Low risk
Świdrak *et al*., 2023	Cross‐sectional	JBI Checklist – Cross‐sectional	Low risk
Świdrak *et al*., 2025	Mixed‐methods	MMAT	Moderate risk
Todd *et al*., 2024	Cross‐sectional	JBI Checklist – Cross‐sectional	Low risk
Valenzuela‐Moguillansky, 2013	Phenomenological qualitative	JBI Checklist – Qualitative	Moderate risk
Valenzuela‐Moguillansky *et al*., 2017	Cross‐sectional	JBI Checklist – Cross‐sectional	Low risk
Van Overmeire *et al*., 2022	Cross‐sectional	JBI Checklist – Cross‐sectional	High risk
Viceconti *et al*., 2022	Cross‐sectional	JBI Checklist – Cross‐sectional	Moderate risk

^†^ *Some concerns* according to Cochrane RoB 2 correspond to a moderate risk of bias.

Cochrane RoB 2, Cochrane Risk of Bias 2; JBI Checklist – Cross‐sectional, JBI Critical Appraisal Checklist for Analytical Cross‐Sectional Studies; JBI Checklist – Qualitative, JBI Critical Appraisal Checklist for Qualitative Research; MMAT, Mixed Methods Appraisal Tool.

Among cross‐sectional studies (*N* = 17), assessed with the JBI Critical Appraisal Checklist, approximately half were rated as low risk of bias, while the remaining studies showed moderate to high risk. The most frequent sources of bias concerned inadequate reporting of participant selection and diagnostic criteria, insufficient control of confounding variables, lack of sample size justification, and suboptimal control group matching.

Of the three randomized controlled trials assessed with the Cochrane Risk of Bias 2 tool, one was judged at low risk, one raised some concerns, and one was rated at high risk, mainly due to issues related to blinding, deviations from intended interventions, and randomization procedures.

Qualitative studies (*N* = 4), evaluated using the JBI checklist for qualitative research, generally presented moderate methodological limitations, with one study rated at high risk of bias. Common weaknesses included limited reflexivity, insufficient transparency in data analysis, and inadequate reporting of strategies to ensure trustworthiness. The only mixed‐methods study showed a moderate risk of bias, primarily due to limited integration of qualitative and quantitative components.

Taken together, although several studies demonstrated acceptable methodological rigor, the overall evidence base is affected by recurrent limitations related to study design, sample characteristics, and methodological transparency, which should be considered when interpreting the findings.

### Meta‐analytic findings

Studies eligible for meta‐analysis are summarized in Table 4. Table 5 presents the main meta‐analytic findings: the multilevel random‐effects meta‐analysis yielded a high pooled effect size across all included studies (*d* = 0.993, *P* < 0.001). The leave‐one‐out analysis revealed that the pooled effect size, its confidence interval, and heterogeneity estimates were not substantially affected by the removal of any single work, suggesting that the results were not driven by individual studies.

**Table 4:** Studies eligible for meta‐analysis

Study	N1 (FM patients)	N2 (Controls)	Effect size (Cohen's d)	SE	Z	*P*‐value	Construct
Akklaya *et al*., 2012	51	41	1.694	0.228	7.430	<0.001	Body image/Body esteem
Berk *et al*., 2020	41	30	0.542	0.232	2.336	<0.05	Body image/Body esteem
	45	30	2.678	0.273	9.810	<0.001	Body image/Body esteem
	55	30	4.563	0.296	15.416	<0.001	Body image/Body esteem
Borg *et al*., 2018	21	21	0.206	0.309	0.667	0.505	Body awareness/interoception
	21	21	1.17	0.34	3.441	<0.001	Body awareness/interoception
Duschek *et al*., 2017	45	31	0.518	0.212	2.443	<0.05	Body awareness/interoception
Martinez *et al*., 2018	14	13	0.61	0.285	2.140	<0.05	Body awareness/interoception
	14	13	0.48	0.278	1.364	0.173	Body image/Body esteem
Schmitz *et al*., 2021	55	55	0.161	0.193	0.834	0.404	Body awareness/interoception
Rost *et al*. 2017	47	45	0.67	0.205	3.268	<0.05	Body awareness/interoception
Valenzuela‐Moguillansky *et al*., 2017	30	29	0.284	0.262	1.084	0.278	Body awareness/interoception

**Table 5:** Main meta‐analytic findings

	Pooled effect size				Residual heterogeneity test		
	Estimate (SE)	*t*	df	*P*	*Q*ₑ	df	*P*
Overall results (BA/I + BI + BE)	0.993 (0.125)	7.923	4.264	0.001	131.257	9	<0.001
Body awareness/interoception	0.470 (0.09)	5.240	4.816	<0.05	9.282	7	0.233
Body image	1.528 (1.391)	1.098	1	0.470	25.440	2	<0.001

BA/I, Body Awareness/Interoception; BE, Body esteem; BI, Body Image.

Figure 2 represents the forest plot summarizing the effect sizes of the included studies. All studies showed a positive effect size, suggesting statistically significant results, although some of their confidence intervals did cross zero.

**Fig. 2:**
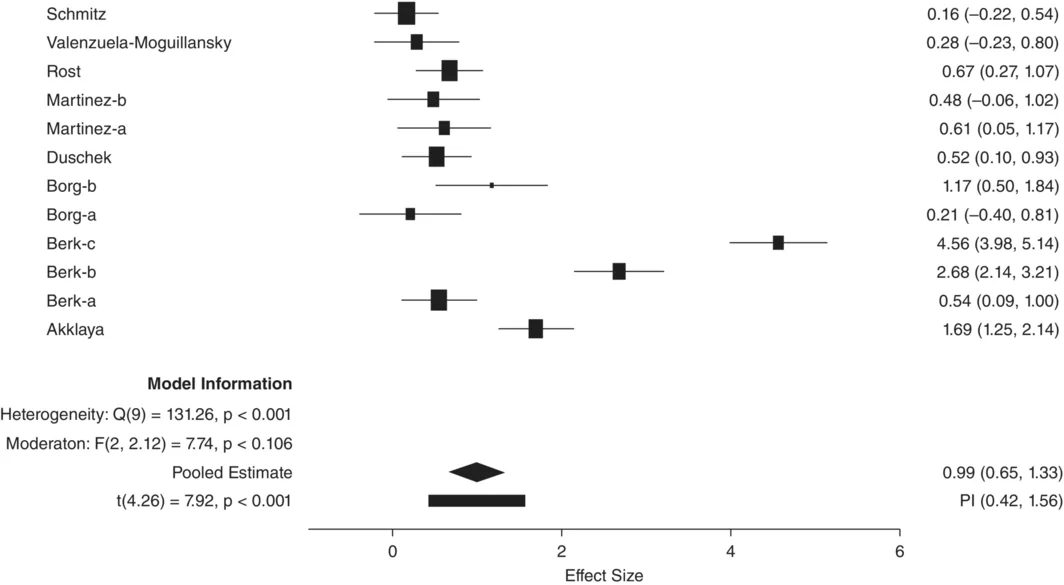
Forest plot of included studies.

Despite these results, the heterogeneity test revealed highly significant variability across studies (*Q* = 131.257, *df* = 11, *P* < 0.001), consistent with a high *I* ^*2*^ (93.1%). Given the limited number of included studies and the high heterogeneity, meta‐analytic and meta‐regression results should be interpreted with caution.

To account for this heterogeneity, we chose to perform a meta‐regression introducing body awareness/interoception and body image as predictors; the construct body esteem was excluded from this analysis because it was represented by a single study (Martínez *et al*., 2018). The omnibus test revealed overall significance of the model (*F*(1, 1.96) = 20.34, *P* = 0.048). When assessed individually, both included constructs showed moderate positive effects, as displayed in Table 6.

**Table 6:** Meta‐regression results

										95% CI	
	*F*	df₁	df_2_	*P*		Estimate (SE)	*t*	df	*P*	Lower	Upper
Construct	20.339	1	1.961	0.048	Intercept	0.474 (0.094)	5.015	4.832	<0.05	0.228	0.719
					Construct (body image)	1.565 (0.347)	4.510	1.961	0.048	0.043	3.087

As Table 7 illustrate, no significant evidence of publication bias was detected using Egger's test (*z* = −1.08, *p* = 0.318); however, the small number of studies may limit the power to detect asymmetry. The results of the meta‐analysis for body awareness/interoception are summarized in Figure 3.

**Table 7:** Funnel plot asymmetry test

	Asymmetry test		Limit estimate		
Estimates	*z*	*P*	Estimate	Lower 95% CI	Upper 95% CI
12	0.998	0.318	−1.084	−5.488	3.320

**Fig. 3:**
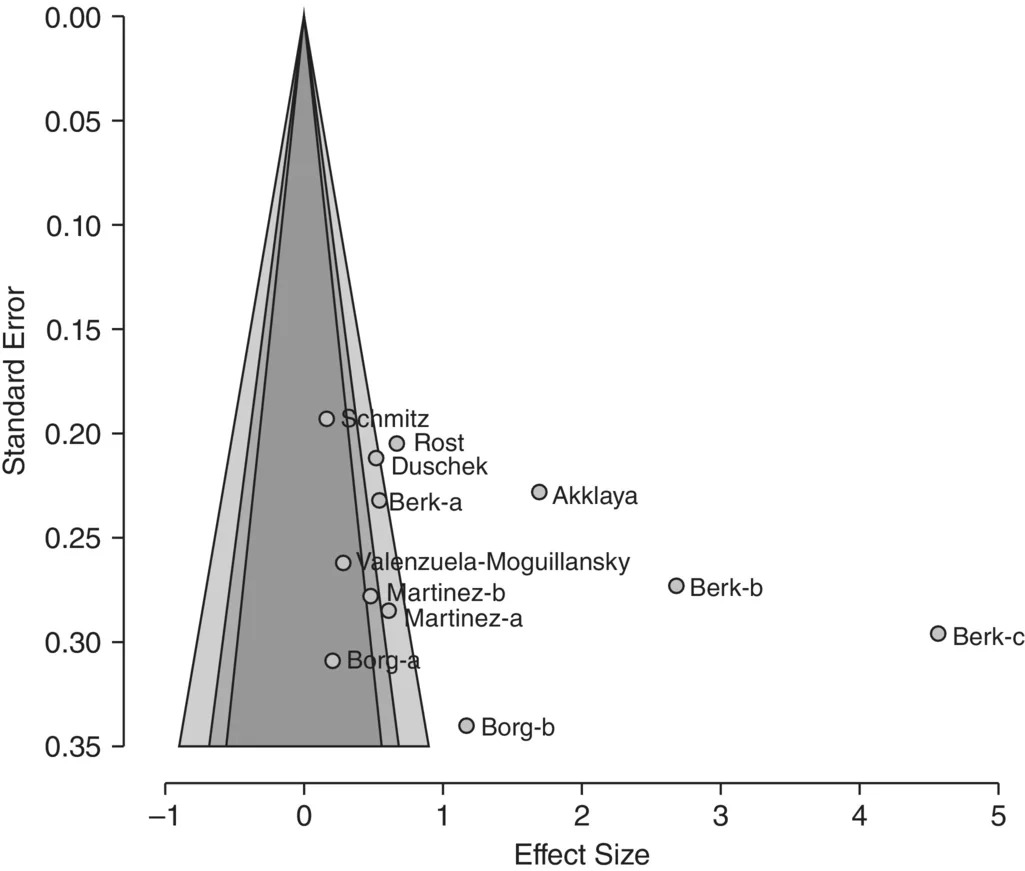
Forest plot for body awareness/interoception.

Given the meta‐regression results, we chose to analyze body awareness/interoception and body image separately through a subgroup analysis: studies assessing the former construct showed no significant heterogeneity (*Qₑ*(7) = 9.28, *p* = 0.233) and a moderate effect size (*d* = 0.47, *P* < 0.05). In contrast, the pooled effect size for body image was statistically insignificant (*d* = 1.53, *p* = 0.47) and showed substantial heterogeneity (*Qₑ*(2) = 25.44, *P* < 0.001). Both depression and anxiety were included as moderators; see Figs 2, 4 for the respective forest plots.

**Fig. 4:**
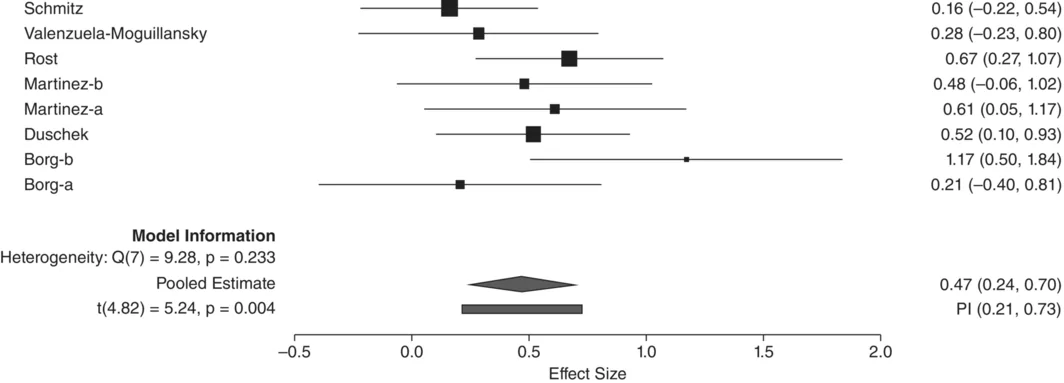
Funnel plot of included studies.

To further explore sources of heterogeneity in the body image domain, we performed a separate meta‐regression that included depression and anxiety as moderators, as they were reported as crucial features of FM patients in all these studies. Depressive symptoms significantly moderate the effect size in a univariate model (*b* = 2.419, *SE* = 0.082, *t*(1, 29) = 29.58, *P* < 0.05); however, when both depression and anxiety were included in a multivariate model, their individual contributions were no longer statistically significant, likely due to high collinearity (see Table 8 and Figure 5).

**Table 8:** Meta‐regression for body image

						95% CI	
	Estimate	Standard error	*t*	df	*P*	Lower	Upper
Intercept	−1.580	1.288	−1.227	1.006	0.434	−17.699	14.540
Depression	2.419	0.082	29.579	1.287	0.009	1.795	3.043
Intercept	−2.424	2.285	−1.061	1.000	0.481	−31.460	26.612
Depression	2.193	0.234	9.373	1.000	0.068	−0.780	5.165
Anxiety	0.507	0.495	1.024	1.000	0.492	−5.787	6.802

## Discussion

This review analyzed 25 studies that investigated different dimensions of the bodily self in FM and provided meta‐analytic findings on 8 studies focused on three specific constructs. Quantitative and qualitative data reported in all included studies, along with our meta‐analytic results, support the existence of recurrent alterations in body image, body schema and perceptual body representation, and body awareness and interoceptive processes, as reported across the included studies (e.g.,^50^, ^54^, ^61^, ^66^). These disorders are not limited to the subjective and phenomenological sphere; FM, in fact, affects both the lived experience of the body and its cognitive and perceptual representations. In particular, evidence from both quantitative (e.g., variability in interoceptive profiles and body image disturbances) and qualitative studies (e.g., the identification of experiential profiles such as the ‘Disconnected Body’) suggests the presence of heterogeneous patterns of bodily self‐alterations rather than a uniform profile.^22^, ^63^ Rather than clearly defined subtypes, these findings point to variability in how bodily disturbances manifest across patients, highlighting the heterogeneity of the condition. This supports the concept of FM as a continuum of symptom severity and somatic‐affective manifestations rather than as a discrete disorder.^69^ Consequently, FM can be interpreted as a spectrum of conditions, encompassing different clinical presentations; this underscores the importance of personalized assessment and intervention strategies.

The literature confirms that body perception is a crucial issue in chronic conditions. In this line, patients who perceive their bodies as ‘manageable’ show better body esteem, particularly in terms of body appreciation.^70^ Conversely, several chronic conditions (e.g., renal failure on dialysis, endometriosis, lupus) are associated with body dissatisfaction and feelings of alienation from one's body,^71^, ^72^, ^73^ indicating that changes in body image should be considered intrinsic characteristics of chronic disease, with a direct negative impact on quality of life. In this regard, our meta‐analytic findings highlight that depressive symptoms may play a pivotal role in shaping these FM patients' perception of their own body, consistent with other works.^3^ The non‐significance of anxiety and depression as moderators of body image in our study when taken together may reflect shared variance between these variables, which are closely related constructs in FM, making it difficult to disentangle their independent contributions. In addition, the limited number of studies and the heterogeneity in anxiety measurement may have further reduced the statistical power of the model (Figs 4, 5).

**Fig. 5:**
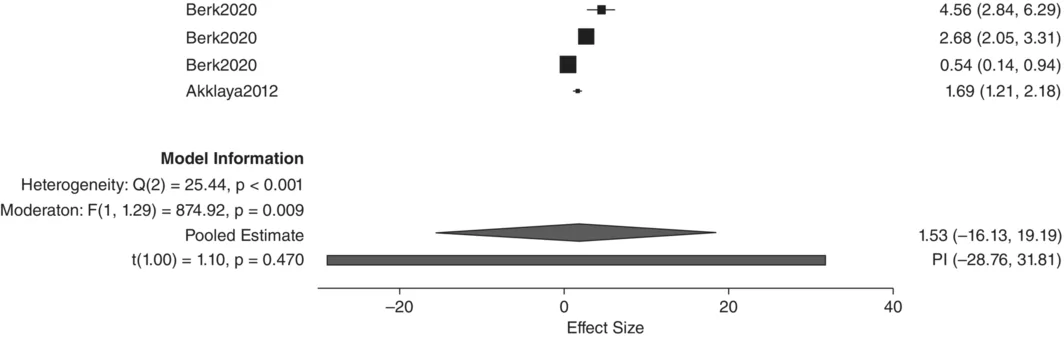
Forest plot for body image.

With regard to interoception, a complex pattern is observed in people with FM, who usually exhibit high scores in ‘noticing’ but lower scores in ‘trusting’, ‘attentional regulation’, and ‘not distracting’,^22^, ^46^, ^62^, ^63^ suggesting heightened attention accompanied by low trust and regulatory difficulties. Importantly, findings across studies suggest a dissociation between different interoceptive dimensions rather than a uniform deficit. While self‐reported interoceptive sensibility is often elevated, objective measures of interoceptive accuracy (e.g., heartbeat detection tasks) yield mixed results, with some studies reporting no differences or even increased accuracy in FM patients. These findings align with a broader picture that highlights reduced interoception accuracy in patients with chronic pain^11^, ^54^, ^74^ and concurrent hypersensitivity to bodily signals.^47^, ^60^ A recent meta‐analysis confirmed that patterns of reduced accuracy and/or increased sensibility are recurring characteristics in patients with chronic pain and may contribute to distortions in body perception and the chronicity of pain.^75^ This combination can be interpreted as a dissociation between the different interoceptive dimensions (i.e., accuracy, sensibility, and awareness)^35^, ^76^, ^77^ with important implications for interoception‐based clinical treatments.^11^ While most of the included studies using the MAIA indicate heightened attention but low trust and regulation in FM patients, one study reported no significant differences compared to healthy controls.^51^ This discrepancy may be related to methodological differences, including variations in sample characteristics, cultural factors, or differences in symptom severity. Such inconsistencies underscore the need for replication studies and careful consideration of contextual factors when interpreting interoceptive profiles in FM.

In addition, meta‐analytic results on body awareness revealed a moderate yet significant impairment in this domain among patients diagnosed with FM compared to healthy controls. Furthermore, the absence of heterogeneity among this cluster of studies may support the idea that interoception and body awareness are partially overlapping dimensions of the bodily self, despite being theoretically distinct – consistent with the definition adopted in this review, in which interoception is understood as a component of the broader construct of body awareness.

As already mentioned, psychological comorbidities such as depression and anxiety are often observed in patients with FM^3^; these disorders further influence interoception. For example, low levels of interoceptive accuracy have been reported in individuals with depression, eating disorders, and somatoform disorders, while high interoceptive sensibility is more frequently associated with symptoms of anxiety.^35^, ^76^ The relationship between the body and pain is also influenced by psychological and emotional factors. Stress, anxiety, and depression, for example, can alter pain transmission pathways and increase sensitivity to nociceptive stimuli. In addition, individuals with chronic pain often report additional somatic symptoms, such as fatigue, sleep disturbances, and cognitive dysfunction, which further complicate the interaction between bodily processes and pain perception. These influences underscore the need to adopt an integrated biopsychosocial perspective when examining chronic pain conditions such as FM.^78^ Stress response systems, such as the hypothalamic–pituitary–adrenal (HPA) axis and the autonomic nervous system, are often dysregulated in patients with chronic pain. These alterations can substantially influence the body's physiological response to pain and contribute to the exacerbation of symptoms.

This dysfunction highlights the complex neurobiological basis of chronic pain and supports the need for research that integrates neuroendocrine and autonomic markers into the study of pain mechanisms and their clinical management.^78^

Overall, evidence shows that interoception in chronic conditions, including FM, is altered and that different subscales directly modulate symptom perception and its impact on quality of life.^12^ Interventions aimed at increasing interoceptive awareness (e.g., mindfulness‐based interventions and body awareness therapies) can improve pain and reduce associated psychological symptoms, such as anxiety and depression.^79^, ^80^ Overall, these findings have important implications for clinical practice and health policy in FM management, particularly regarding the need to integrate systematic assessment of bodily self‐disturbances (including interoception, body image, body schema, and perceptual body representation) into multidisciplinary evaluation protocols and to develop targeted, individualized interventions addressing these dimensions within a biopsychosocial framework.

### Alterations of the Bodily Self in Fibromyalgia: Mechanisms and Manifestations

Alterations in bodily self‐perception in chronic pain, and therefore also in FM, could result from maladaptive neuroplasticity processes involving bodily representation networks.^22^, ^81^, ^82^, ^83^, ^84^ Central sensitization represents a key mechanism that amplifies neural transmission and contributes to an increased salience of bodily signals, rather than a uniform increase in interoceptive accuracy.^64^, ^85^ In this framework, bodily hypervigilance in FM refers to a persistent and excessive monitoring of internal (e.g., visceral, proprioceptive) and external (e.g., tactile, environmental) bodily sensations, which may underlie the amplified perception of both painful and non‐painful stimuli.^60^, ^86^ Such heightened attention to bodily cues can exacerbate symptom awareness and distress, thereby sustaining central sensitization mechanisms over time. Furthermore, increased body schema plasticity^47^, ^58^ would reflect changes in somatosensory, parietal, and insular areas, while neuroimaging studies have shown alterations in the thalamus, anterior cingulate cortex, and insula associated with increased sensitivity to pain, stress, and catastrophizing.^87^, ^88^, ^89^, ^90^ FM patients' difficulties in motor imagery and kinesthetic representation may be modulated by symptom duration and disease severity. Longer disease history and higher symptom intensity appear to increase the likelihood of developing alterations in the mental representation of action, suggesting that prolonged nociceptive input may exacerbate disruptions in body schema and sensorimotor integration.^61^ This highlights the progressive nature of body schema disturbances in FM and underscores the importance of early interventions targeting body awareness and motor imagery.

The relationship between pain and the parietal cortex is complex and multifaceted, with important implications for chronic pain conditions such as FM. The parietal cortex plays a central role in integrating sensory information from different modalities, contributing to the construction and updating of both body schema and perceptual body representation. Alterations in this integrated capacity may help explain why patients with FM often report amplified and poorly localized pain, as well as difficulty distinguishing painful from non‐painful sensations. This altered sensory processing is observable in the phenomenon of allodynia, in which normally non‐painful stimuli are perceived as painful.^91^ In addition to sensory integration, the parietal cortex contributes to modulation processes through which contextual factors, expectations, and body‐related feedback influence pain perception. This is particularly relevant in FM, where heightened attentional focus on bodily sensations and disturbances in body schema and perceptual body representation have been consistently observed. Clinical evidence further shows that parietal dysfunction may lead to atypical pain‐related experiences, including distortions in body awareness and pain asymbolia, which mirror phenomena frequently described in chronic pain syndromes. Taken together, these findings highlight the parietal cortex as a crucial target for understanding the mechanisms underlying pain amplification in FM and suggest that interventions aimed at improving body awareness and recalibrating body schema and perceptual body representation may hold therapeutic potential for this patient population.^88^

These data also seem to support the bidirectional relationship between pain and body representations. For instance, the sense of ownership can modulate the painful experience, generating either analgesic or hyperalgesic effects, while pain compromises motor imagery in the affected areas.^92^

On a clinical‐functional level, multisystemic dysregulation affects both afferent and efferent circuits.^93^, ^94^, ^95^ Fear of pain promotes avoidance behaviors that alter motor patterns, reducing agility and coordination and leading to a progressive loss of body confidence, as confirmed by the negative correlation between the ‘trusting’ dimension of the MAIA and the impact of the disease.^66^ The involvement of small nerve fibers also appears to be crucial. Notably, body perception disorders are associated with symptoms of small fiber neuropathy, which affects about half of FM patients.^18^, ^96^ At the same time, interoception presents paradoxical characteristics. Patients report high awareness of bodily sensations but poor functional confidence, with difficulty detaching themselves from somatic perceptions and regulating their attention.^68^, ^97^ Emotional‐cognitive factors, such as depression, anxiety, catastrophizing, and alexithymia, seem to function as both antecedents and consequences of bodily self‐alterations, fueling vicious cycles that maintain and aggravate symptoms.^3^, ^60^, ^62^ From this perspective, interoceptive impairment plays a central role in emotional regulation. According to the somatic marker hypothesis,^98^ physiological representations contribute to behavioral adaptation, but poor interoceptive awareness reduces control over the body and increases hypochondriacal concerns.^54^, ^98^

Furthermore, excessive attention to symptoms amplifies psychological distress, further reinforcing this cycle.^62^, ^99^ This excessive monitoring of symptoms reflects bodily hypervigilance, whereby individuals pay disproportionate attention to bodily sensations and potential signs of threat.^86^, ^100^ In FM, this hypervigilant attitude may amplify the salience and subjective intensity of bodily sensations and contribute to distorted body awareness, thus maintaining the vicious cycle between pain and emotional distress. Furthermore, according to the generalized hypervigilance hypothesis (GHH), individuals with FM tend to devote disproportionate attention not only to pain‐related stimuli but also to a wide range of sensory events, such as light, sound, touch, and temperature changes. This increased attention may amplify the salience of painful and non‐painful sensations, leading to an intensified perception of bodily experiences. As a consequence, patients may become more prone to maladaptive emotional and behavioral responses, such as avoidance, catastrophizing, and increased functional impairment. The GHH therefore provides a valuable framework for understanding how attentional biases toward sensory input could contribute to the maintenance and exacerbation of symptomatology in FM.^60^

Embodiment also shows paradoxical aspects. In fact, higher levels of perceptual alterations predict a greater degree of embodiment toward external stimuli,^22^ and the disruption of implicit knowledge of body topography – which can be understood as a disruption of body schema – can generate the sensation of pain without perceiving it as one's own,^65^ signaling a brain–body desynchronization typical of FM.^101^ Body distortions, while constituting a form of adaptive plasticity that facilitates the incorporation of virtual bodies,^102^, ^103^ tend to persist even when pain improves, suggesting a strong emotional‐affective component independent of nociception.^47^, ^58^, ^104^ Qualitative evidence supports this interpretation: patients frequently describe their bodies as alienated, unreliable, or disconnected from the self,^53^, ^55^ and the persistence of these experiences – even in the absence of acute pain – underscores the affective and identity‐related dimensions of bodily self‐disturbance in FM. Despite this, they remain susceptible to cognitive‐behavioral interventions aimed at improving body awareness and restructuring body image,^105^, ^106^ in line with the dynamic and reciprocal nature of the interactions between pain and body representations.^92^

These findings carry specific implications for intervention, which can be differentiated according to the three domains examined in this review. For body image and body esteem, cognitive‐behavioral and acceptance‐based approaches targeting maladaptive body‐related cognitions and evaluative attitudes appear particularly suited, given the affective and identity‐related dimensions of body image disturbance documented in FM.^105^ For body schema and perceptual body representation, sensory re‐education, graded motor imagery, and virtual reality‐based embodiment paradigms represent promising avenues, given FM patients' documented susceptibility to bodily illusions and altered multisensory integration.^9^, ^22^ For body awareness and interoception, mindfulness‐based and body awareness therapies have demonstrated efficacy in improving interoceptive sensibility and reducing hypervigilance in chronic pain populations^79^ and appear particularly suited to the paradoxical interoceptive profile observed in FM, characterized by heightened noticing alongside reduced trusting and attentional regulation. Given the high comorbidity between bodily self‐disturbances and psychological symptoms in FM, multimodal interventions integrating somatic, cognitive, and emotional components are likely to be most effective.

In addition, given the affective dimension underlying body distortions, therapeutic approaches explicitly targeting this component are warranted. Emotion‐focused strategies could contribute to reducing distress associated with altered bodily self‐perception and fostering a more compassionate relationship with one's body, thereby improving overall quality of life in FM.

### Limitations and Future Directions

It is necessary to acknowledge some limitations of this review. The literature has limitations, including the predominance of cross‐sectional designs, small and clinically heterogeneous samples, and the use of assessment tools that are not always validated for populations affected by FM. Furthermore, despite an overall sufficient number of studies for a qualitative synthesis, quantitative evidence remains heterogeneous across different areas of body image and has limited the scope of quantitative analyses and highlights important gaps in the literature. The homogeneity of samples further limits validity, as most studies predominantly involved female, white, and Western participants.

Furthermore, the frequent use of healthy control groups limits the specificity of the alterations observed; the inclusion of comparison groups with other chronic pain conditions would help clarify whether body image disturbances are specific to FM or represent transdiagnostic characteristics of chronic pain.

Another relevant limitation concerns the widespread use of self‐assessment measures which, while essential for capturing subjective experience, may be influenced by social desirability and cultural norms. Integrating subjective assessments with objective or clinician‐rated measures would help improve interpretability.

Future studies should prioritize longitudinal designs and mixed methods to investigate the processes linking FM symptoms and bodily self‐disorders. Combining phenomenological data with neurobiological markers (e.g., neuroimaging, autonomic and small fiber measures) may clarify underlying mechanisms and support the identification of clinically meaningful biomarkers. Finally, the development and standardization of sensitive and FM‐specific tools, along with the evaluation of targeted interventions such as embodiment‐based therapies, sensory re‐education, and interoceptive regulation, will be critical to improving both the quality of research and patient‐centered clinical practice.

## Conclusion

This systematic review highlights how FM involves widespread alterations of the bodily self, affecting body image and body esteem, body schema and perceptual body representation, and body awareness and interoceptive functioning. These disturbances manifest both subjectively, through experiences of estrangement and alienation from the body, and objectively, through experimental tasks documenting difficulties in multisensory and representational integration. The body of evidence suggests that FM is not only a painful syndrome but also a condition that profoundly compromises the way people perceive and experience their bodies.

From a clinical perspective, these alterations require therapeutic interventions that go beyond pain reduction and include strategies aimed at improving body awareness and promoting interoceptive regulation. Integrated interventions, such as sensory training, embodiment‐based therapies, interoceptive approaches, and body‐centered psychotherapies, may represent promising options.

Future longitudinal and multicenter studies, integrating subjective and neurobiological measures, will be crucial for a deeper understanding of the underlying mechanisms and the development of personalized clinical protocols.

## Author contributions

Martina Mesce: Conceptualization; Data curation; Formal analysis; Investigation; Methodology; Resources; Validation; Visualization; Roles/Writing – original draft; Writing – review & editing. Matteo Scalzeri: Conceptualization; Data curation; Formal analysis; Investigation; Methodology; Resources; Validation; Visualization; Roles/Writing – original draft; Writing – review & editing. Carmen M. Galvez‐Sánchez: Conceptualization; Formal analysis; Resources; Project administration; Supervision; Validation; Visualization; Writing – review & editing; Funding acquisition. Julio A. Camacho‐Ruiz: Conceptualization; Data curation; Formal analysis; Investigation; Methodology; Resources; Validation; Visualization; Roles/Writing – original draft; Writing – review & editing. Rosa M. Limiñana‐Gras: Conceptualization; Data curation; Formal analysis; Investigation; Methodology; Resources; Validation; Visualization; Roles/Writing – original draft; Writing – review & editing. Federica Galli: Conceptualization; Formal analysis; Methodology; Resources; Project administration; Supervision; Validation; Visualization; Roles/Writing – original draft; Writing – review & editing; Funding acquisition. All authors have read and agreed to the published version of the manuscript.

## Disclosure statement

The authors declare no conflicts of interest.

## Supporting information


**Data S1** Supporting information.

## Data Availability

The data that support the findings of this study are available from the corresponding author upon reasonable request.

## References

[1] Clauw DJ . Fibromyalgia: A clinical review. JAMA 2014; 311: 1547–1555. 10.1001/jama.2014.3266 24737367

[2] Cohen H . Controversies and challenges in fibromyalgia: A review and a proposal. Ther. Adv. Musculoskelet. Dis. 2017; 9: 115–127. 10.1177/1759720X17699199 PMC5394529 28458723

[3] Galvez‐Sánchez CM , Duschek S , Reyes Del Paso GA . Psychological impact of fibromyalgia: Current perspectives. Psychol. Res. Behav. Manag. 2019; 12: 117–127. 10.2147/PRBM.S178240 PMC6386210 30858740

[4] Häuser W , Ablin J , Fitzcharles MA *et al*. Fibromyalgia. Nat. Rev. Dis. Primers. 2015; 1: 15022. 10.1038/nrdp.2015.22 27189527

[5] Fitzcharles MA , Brachaniec M , Cooper L *et al*. A paradigm change to inform fibromyalgia research priorities by engaging patients and health care professionals. Can. J. Pain 2017; 1: 137–147. 10.1080/24740527.2017.1374820 PMC8730558 35005349

[6] Gokcen N , Coskun Benlidayi I , Tamam L , Demirkol ME , Yesiloglu C , Guzel R . Type D personality and self‐esteem in patients with fibromyalgia: A cross‐sectional case–control study. Rheumatol. Int. 2022; 42: 1027–1034. 10.1007/s00296-022-05118-z 35353202

[7] Levy S , Ohayon S , Avitsur R , Geller S . Psychological distress in women with fibromyalgia: The roles of body appreciation, self‐compassion, and self‐criticism. Int. J. Behav. Med. 2025; 32: 351–359. 10.1007/s12529-024-10302-5 PMC12134045 38886330

[8] CS MC , Haigh RC , Shenker NG , Lewis J , Blake DR . Phantoms in rheumatology. In: Osteoarthritic Joint Pain [Internet]. John Wiley & Sons, Ltd, Hoboken, NJ, US, 2004 [cited 2026 Jan 21]; 154–178. 10.1002/0470867639.ch11 15283449

[9] Moseley GL . Distorted body image in complex regional pain syndrome. Neurology 2005; 65: 773. 10.1212/01.wnl.0000174515.07205.11 16157921

[10] Berntson GG , Khalsa SS . Neural circuits of Interoception. Trends Neurosci. 2021; 44: 17–28. 10.1016/j.tins.2020.09.011 PMC8054704 33378653

[11] Di Lernia D , Serino S , Riva G . Pain in the body. Altered interoception in chronic pain conditions: A systematic review. Neurosci. Biobehav. Rev. 2016; 71: 328–341. 10.1016/j.neubiorev.2016.09.015 27654341

[12] Locatelli G , Matus A , James R *et al*. What is the role of interoception in the symptom experience of people with a chronic condition? A systematic review. Neurosci. Biobehav. Rev. 2023; 148: 105142. 10.1016/j.neubiorev.2023.105142 36965864

[13] Galli F , Nimbi FM , Renzi A *et al*. Best practice recommendations for clinical psychological interventions in patients with fibromyalgia. Clin. Exp. Rheumatol. 2025; 43: 1112–1121. 10.55563/clinexprheumatol/4arpi4 40470546

[14] Macfarlane GJ , Kronisch C , Dean LE *et al*. EULAR revised recommendations for the management of fibromyalgia. Ann. Rheum. Dis. 2017; 76: 318–328. 10.1136/annrheumdis-2016-209724 27377815

[15] Schilder P , Wertman R . A imagem do corpo. In: A Imagem Do Corpo [Internet]. Martins Fontes, São Paulo, Brazil, 1994 [cited 2026 Apr 22]; 316 Available from: https://pesquisa.bvsalud.org/portal/resource/pt/biblio-1085789.

[16] Cash TF . Body image: Past, present, and future. Body Image 2004; 1: 1–5. 10.1016/S1740-1445(03)00011-1 18089136

[17] Peres RS , de F CS , dos SMA . Subjective aspects of body image in women with fibromyalgia. J. Hum. Growth Dev. 2020; 30: 425–433.

[18] Viceconti A , Geri T , De Luca S , Maselli F , Rossettini G , Testa M . Body perception distortions correlate with neuropathic features in Italian fibromyalgic patients: Findings from a self‐administered online survey. Musculoskelet. Sci. Pract. 2022; 60: 102570. 10.1016/j.msksp.2022.102570 35594609

[19] Grogan S . Body image and health: Contemporary perspectives. J. Health Psychol. 2006; 11: 523–530. 10.1177/1359105306065013 16769732

[20] Lotze M , Moseley GL . Role of distorted body image in pain. Curr. Rheumatol. Rep. 2007; 9: 488–496. 10.1007/s11926-007-0079-x 18177603

[21] Thompson JK . Assessing body image disturbance: Measures, methodology, and implementation. In: Body Image, Eating Disorders, and Obesity: An Integrative Guide for Assessment and Treatment. American Psychological Association, Washington, DC, US, 2001; 49–81.

[22] Świdrak J , Arias A , de la Calle ER , Collado Cruz A , Sanchez‐Vives MV . Virtual embodiment in fibromyalgia. Sci. Rep. 2023; 13: 10719. 10.1038/s41598-023-36861-3 PMC10318080 37400503

[23] Gallagher S . Body image and body schema: A conceptual clarification. J. Mind Behav. 1986; 7: 541–554.

[24] Pitron V , Alsmith A , de Vignemont F . How do the body schema and the body image interact? Conscious. Cogn. 2018; 65: 352–358. 10.1016/j.concog.2018.08.007 30262229

[25] Raoul L , Grosbras MH . Relating different dimensions of bodily experiences: Review and proposition of an integrative model relying on phenomenology, predictive brain and neuroscience of the self. Neurosci. Biobehav. Rev. 2023; 148: 105141. 10.1016/j.neubiorev.2023.105141 36965863

[26] Berlucchi G , Aglioti S . The body in the brain: Neural bases of corporeal awareness. Trends Neurosci. 1997; 20: 560–564. 10.1016/s0166-2236(97)01136-3 9416668

[27] Bray H , Moseley GL . Disrupted working body schema of the trunk in people with back pain. Br. J. Sports Med. 2011; 45: 168–173. 10.1136/bjsm.2009.061978 19887441

[28] Schwoebel J , Friedman R , Duda N , Coslett HB . Pain and the body schema: Evidence for peripheral effects on mental representations of movement. Brain 2001; 124: 2098–2104. 10.1093/brain/124.10.2098 11571225

[29] Schwoebel J , Coslett HB , Bradt J , Friedman R , Dileo C . Pain and the body schema: Effects of pain severity on mental representations of movement. Neurology 2002; 59: 775–777. 10.1212/wnl.59.5.775 12221179

[30] Craig AD . How do you feel? Interoception: The sense of the physiological condition of the body. Nat. Rev. Neurosci. 2002; 3: 655–666. 10.1038/nrn894 12154366

[31] Khalsa SS , Adolphs R , Cameron OG *et al*. Interoception and mental health: A roadmap. Biol. Psychiatry Cogn. Neurosci. Neuroimaging 2018; 3: 501–513. 10.1016/j.bpsc.2017.12.004 PMC6054486 29884281

[32] Garfinkel SN , Seth AK , Barrett AB , Suzuki K , Critchley HD . Knowing your own heart: Distinguishing interoceptive accuracy from interoceptive awareness. Biol. Psychol. 2015; 104: 65–74. 10.1016/j.biopsycho.2014.11.004 25451381

[33] Farb N , Daubenmier J , Price CJ *et al*. Interoception, contemplative practice, and health. Front. Psychol. 2015; 6: 763. 10.3389/fpsyg.2015.00763. PMC4460802 26106345

[34] Suksasilp C , Garfinkel SN . Towards a comprehensive assessment of interoception in a multi‐dimensional framework. Biol. Psychol. 2022; 168: 108262. 10.1016/j.biopsycho.2022.108262 35026353

[35] Pollatos O , Kurz AL , Albrecht J *et al*. Reduced perception of bodily signals in anorexia nervosa. Eat. Behav. 2008; 9: 381–388. 10.1016/j.eatbeh.2008.02.001 18928900

[36] Terhaar J , Viola FC , Bär KJ , Debener S . Heartbeat evoked potentials mirror altered body perception in depressed patients. Clin. Neurophysiol. 2012; 123: 1950–1957. 10.1016/j.clinph.2012.02.086 22541740

[37] Weiss S , Sack M , Henningsen P , Pollatos O . On the interaction of self‐regulation, Interoception and pain perception. Psychopathology 2014; 47: 377–382. 10.1159/000365107 25342240

[38] Tsakiris M , Critchley H . Interoception beyond homeostasis: Affect, cognition and mental health. Philos. Trans. R. Soc. Lond. B Biol. Sci. 2016; 371: 20160002. 10.1098/rstb.2016.0002 PMC5062092 28080961

[39] Page MJ , JE MK , Bossuyt PM *et al*. The PRISMA 2020 statement: An updated guideline for reporting systematic reviews. BMJ 2021; 372: n71. 10.1136/bmj.n71 PMC8005924 33782057

[40] Stern C , Kleijnen J . Language bias in systematic reviews: You only get out what you put in. JBI Evid. Synth. 2020; 18: 1818–1819. 10.11124/JBIES-20-00361 32925418

[41] Munn Z , Barker TH , Moola S *et al*. Methodological quality of case series studies: An introduction to the JBI critical appraisal tool. JBI Evid. Synth. 2020; 18: 2127–2133. 10.11124/JBISRIR-D-19-00099 33038125

[42] JAC S , Savović J , Page MJ *et al*. RoB 2: A revised tool for assessing risk of bias in randomised trials. BMJ 2019; 366: l4898. 10.1136/bmj.l4898 31462531

[43] Lockwood C , Porritt K , Munn Z *et al*. Chapter 2: Systematic reviews of qualitative evidence. In: In: Aromataris E, Munn Z, Editors. The Joanna Briggs Institute, Adelaide, South Australia, Australia, 2017 [cited 2026 Jan 21]. Available from: http://wiki.joannabriggs.org/display/MANUAL/Chapter+2:+Systematic+reviews+of+qualitative+evidence.

[44] Hong QN , Fàbregues S , Bartlett G *et al*. The mixed methods appraisal tool (MMAT) version 2018 for information professionals and researchers. Educ. Inform. 2018; 34: 285–291.

[45] Berk E , BF Koçyi̇ği̇t , Berk ŞŞ . The relationship between disease severity and body perception, mood disorder and quality of life in fibromyalgia syndrome. Turk. Klin. J. Med. Sci. 2020; 40: 154–160.

[46] Borg C , Chouchou F , Dayot‐Gorlero J *et al*. Pain and emotion as predictive factors of interoception in fibromyalgia. J. Pain Res. 2018; 11: 823–835. 10.2147/JPR.S152012 PMC5914549 29719416

[47] Martínez E , Aira Z , Buesa I , Aizpurua I , Rada D , Azkue JJ . Embodied pain in fibromyalgia: Disturbed somatorepresentations and increased plasticity of the body schema. PLoS One 2018; 13: e0194534. 10.1371/journal.pone.0194534 PMC5889164 29624596

[48] Agostinho M , Capelas ML , Pimentel‐Santos FM , Canaipa R . Disentangling interoception and its links to cognitive functioning in fibromyalgia. Sci. Rep. 2025; 15: 31403. 10.1038/s41598-025-15087-5 PMC12381188 40858714

[49] Akkaya N , Akkaya S , Atalay NS , Balci CS , Sahin F . Relationship between the body image and level of pain, functional status, severity of depression, and quality of life in patients with fibromyalgia syndrome. Clin. Rheumatol. 2012; 31: 983–988. 10.1007/s10067-012-1965-9 22395855

[50] Augière T , Metral M , Simoneau M , Mercier C . Preserved tactile distance estimation despite body representation distortions in individuals with fibromyalgia. Front. Pain Res. 2024; 5: 1414927. 10.3389/fpain.2024.1414927. PMC11306202 39119526

[51] Borg C , Emond FC , Colson D , Laurent B , Michael GA . Attentional focus on subjective interoceptive experience in patients with fibromyalgia. Brain Cogn. 2015; 101: 35–43. 10.1016/j.bandc.2015.10.002 26562055

[52] JEA B , Schoster B , Callahan LF . Comparisons of body image perceptions of a sample of black and white women with rheumatoid arthritis and fibromyalgia in the US. Open Rheumatol. J. 2015; 9: 1–7. 10.2174/1874312901409010001 PMC4319189 25674181

[53] Calsius J , Courtois I , Stiers J , Bie JD . How do fibromyalgia patients with alexithymia experience their body? A qualitative approach. SAGE Open 2015; 5: 2158244015574631.

[54] Duschek S , Montoro CI , Reyes del Paso GA . Diminished interoceptive awareness in fibromyalgia syndrome. Behav. Med. 2017; 43: 100–107. 10.1080/08964289.2015.1094442 26431269

[55] Horwitz EB , Theorell T , Anderberg UM . Fibromyalgia patients' own experiences of video self‐interpretation: A phenomenological‐hermeneutic study. Scand. J. Caring Sci. 2003; 17: 257–264. 10.1046/j.1471-6712.2003.00223.x 12919460

[56] Kircali S , Özcan ÖÖ , Karahan M . Pain neuroscience education and motor imagery‐based exercise protocol for patients with fibromyalgia: A randomized controlled trial. Brain Behav. 2024; 14: e70013. 10.1002/brb3.70013 PMC11391019 39262170

[57] Martínez E , Guillen V , Buesa I , Azkue JJ . A distorted body schema and susceptibility to experiencing anomalous somatosensory sensations in fibromyalgia syndrome. Clin. J. Pain 2019; 35: 887–893. 10.1097/AJP.0000000000000754 31433318

[58] Martínez E , Buesa I , Guillén V , Azkue JJ . Short‐term improvements in the body schema can modulate pain perception in fibromyalgia syndrome. Clin. Exp. Rheumatol. 2022; 40: 1127–1135. 10.55563/clinexprheumatol/2vn0uh 35349412

[59] Menten LA , Franco KFM , Franco YRS , Miyamoto GC , Reis FJJ , Cabral CMN . Do patients with fibromyalgia have body image and tactile acuity distortion? Pain Pract. 2022; 22: 678–687. 10.1111/papr.13153 36345889

[60] Rost S , DML VR , Schulz A , Crombez G , Vögele C . Generalized hypervigilance in fibromyalgia: Normal interoceptive accuracy, but reduced self‐regulatory capacity. J. Psychosom. Res. 2017; 93: 48–54. 10.1016/j.jpsychores.2016.12.003 28107892

[61] Scandola M , Pietroni G , Landuzzi G , Polati E , Schweiger V , Moro V . Bodily illusions and motor imagery in fibromyalgia. Front. Hum. Neurosci. 2022; 15: 798912. 10.3389/fnhum.2021.798912. PMC8811121 35126075

[62] Schmitz N , Napieralski J , Schroeder D , Loeser J , Gerlach AL , Pohl A . Interoceptive sensibility, alexithymia, and emotion regulation in individuals suffering from fibromyalgia. Psychopathology 2021; 54: 144–149. 10.1159/000513774 33951648

[63] Świdrak J , Rodriguez T , Polino L , Arias A , Torres X , Sanchez‐Vives MV . Drawing the lines of fibromyalgia: A mixed‐methods approach to mapping body image, body schema, and emotions in patient subtypes. Psychol. Health Med. 2025; 30: 615–635. 10.1080/13548506.2024.2424997 39557041

[64] Todd J , Plans D , Lee MC *et al*. Heightened interoception in adults with fibromyalgia. Biol. Psychol. 2024; 186: 108761. 10.1016/j.biopsycho.2024.108761 38309512

[65] Valenzuela‐Moguillansky C . Pain and body awareness. An exploration of the bodily experience of persons suffering from fibromyalgia. Constr. Found. 2013; 8: 339–350.

[66] Valenzuela‐Moguillansky C , Reyes‐Reyes A , Gaete MI . Exteroceptive and interoceptive body‐self awareness in fibromyalgia patients. Front. Hum. Neurosci. 2017; 11: 117. 10.3389/fnhum.2017.00117. PMC5346579 28348526

[67] Van Overmeire R , Vesentini L , Vanclooster S , Muysewinkel E , Bilsen J . Body image, medication use, and mental health among women with fibromyalgia in Flanders, Belgium. Int. J. Environ. Res. Public Health 2022; 19: 1418. 10.3390/ijerph19031418 PMC8835128 35162441

[68] Mehling WE , Price C , Daubenmier JJ , Acree M , Bartmess E , Stewart A . The multidimensional assessment of interoceptive awareness (MAIA). PLoS One 2012; 7: e48230. 10.1371/journal.pone.0048230 PMC3486814 23133619

[69] Wolfe F . Fibromyalgianess. Arthritis Care Res. 2009; 61: 715–716. 10.1002/art.24553 19479689

[70] Markey CH , Dunaev JL , August KJ . Body image experiences in the context of chronic pain: An examination of associations among perceptions of pain, body dissatisfaction, and positive body image. Body Image 2020; 32: 103–110. 10.1016/j.bodyim.2019.11.005 31862524

[71] Melis I , Litta P , Nappi L , Agus M , Melis GB , Angioni S . Sexual function in women with deep endometriosis: Correlation with quality of life, intensity of pain, depression, anxiety, and body image. Int. J. Sex. Health 2015; 27: 175–185.

[72] Öyekçin DG , Gülpek D , Sahin EM , Mete L . Depression, anxiety, body image, sexual functioning, and dyadic adjustment associated with dialysis type in chronic renal failure. Int. J. Psychiatry Med. 2012; 43: 227–241. 10.2190/PM.43.3.c 22978081

[73] Rodrigues L , Sim‐Sim MMF , Sousa L , Faria‐Schützer DB , Surita FG . Self‐concept and body image of people living with lupus: A systematic review. Int. J. Rheum. Dis. 2021; 24: 1339–1353. 10.1111/1756-185X.14187 34314107

[74] Solcà M , Park HD , Bernasconi F , Blanke O . Behavioral and neurophysiological evidence for altered interoceptive bodily processing in chronic pain. Neuroimage 2020; 217: 116902. 10.1016/j.neuroimage.2020.116902 32438047

[75] Horsburgh A , Summers SJ , Lewis A , Keegan RJ , Flood A . The relationship between pain and Interoception: A systematic review and meta‐analysis. J. Pain 2024; 25: 104476. 10.1016/j.jpain.2024.01.341 38244898

[76] Dunn BD , Stefanovitch I , Evans D , Oliver C , Hawkins A , Dalgleish T . Can you feel the beat? Interoceptive awareness is an interactive function of anxiety‐ and depression‐specific symptom dimensions. Behav. Res. Ther. 2010; 48: 1133–1138. 10.1016/j.brat.2010.07.006 PMC2964892 20692645

[77] Zoellner LA , Craske MG . Interoceptive accuracy and panic. Behav. Res. Ther. 1999; 37: 1141–1158. 10.1016/s0005-7967(98)00202-2 10596462

[78] Crofford LJ . Chronic pain: Where the body meets the brain. Trans. Am. Clin. Climatol. Assoc. 2015; 126: 167–183. PMC4530716 26330672

[79] Courtois I , Cools F , Calsius J . Effectiveness of body awareness interventions in fibromyalgia and chronic fatigue syndrome: A systematic review and meta‐analysis. J. Bodyw. Mov. Ther. 2015; 19: 35–56. 10.1016/j.jbmt.2014.04.003 25603742

[80] Varkey E , Gottfridsson R , Grimby‐EKMAN A , Bjarnegård Sellius A , Östman M , Andréll P . Body awareness, stress and symptoms in autonomic dysfunction in patients with chronic pain: An explorative study. J. Rehabil. Med. Clin. Commun. 2024; 7: 13374. 10.2340/jrmcc.v7.13374 PMC11218755 38957475

[81] Cavicchioli M , Scalabrini A , Nimbi F *et al*. Fibromyalgia and the painful self: A meta‐analysis of resting‐state fMRI data. J. Psychiatr. Res. 2025; 183: 61–71. 10.1016/j.jpsychires.2025.01.048 39938202

[82] Cauda F , Palermo S , Costa T *et al*. Gray matter alterations in chronic pain: A network‐oriented meta‐analytic approach. Neuroimage Clin. 2014; 4: 676–686. 10.1016/j.nicl.2014.04.007 PMC4053643 24936419

[83] Kuner R , Flor H . Structural plasticity and reorganisation in chronic pain. Nat. Rev. Neurosci. 2016; 18: 20–30. 10.1038/nrn.2016.162 27974843

[84] Pujol J , Blanco‐Hinojo L , Doreste A *et al*. Distinctive alterations in the functional anatomy of the cerebral cortex in pain‐sensitized osteoarthritis and fibromyalgia patients. Arthritis Res. Ther. 2022; 24: 252. 10.1186/s13075-022-02942-3 PMC9650905 36369217

[85] Woolf CJ . Central sensitization: Implications for the diagnosis and treatment of pain. Pain 2011; 152: S2–S15. 10.1016/j.pain.2010.09.030 PMC3268359 20961685

[86] Van Damme S , Van Hulle L , Spence C , Devulder J , Brusselmans G , Crombez G . Hypervigilance for innocuous tactile stimuli in patients with fibromyalgia: An experimental approach. Eur. J. Pain 2015; 19: 706–714. 10.1002/ejp.593 25252089

[87] Ceko M , Bushnell MC , Fitzcharles MA , Schweinhardt P . Fibromyalgia interacts with age to change the brain. Neuroimage Clin. 2013; 3: 249–260. 10.1016/j.nicl.2013.08.015 PMC3814958 24273710

[88] Gallace A , Bellan V . The parietal cortex and pain perception: A body protection system. Handb. Clin. Neurol. 2018; 151: 103–117. 10.1016/B978-0-444-63622-5.00005-X 29519454

[89] Lazaridou A , Kim J , Cahalan CM *et al*. Effects of cognitive‐behavioral therapy (CBT) on brain connectivity supporting catastrophizing in fibromyalgia. Clin. J. Pain 2017; 33: 215–221. 10.1097/AJP.0000000000000422 PMC5296218 27518491

[90] Lutz J , Jäger L , de Quervain D *et al*. White and gray matter abnormalities in the brain of patients with fibromyalgia: A diffusion‐tensor and volumetric imaging study. Arthritis Rheum. 2008; 58: 3960–3969. 10.1002/art.24070 19035484

[91] Staud R , Godfrey MM , Stroman PW . Fibromyalgia is associated with hypersensitivity but not with abnormal pain modulation: Evidence from QST trials and spinal fMRI. Front. Pain Res. 2023; 4: 1284103. 10.3389/fpain.2023.1284103. PMC10728773 38116188

[92] Beccherle M , Scandola M . How pain and body representations transform each other: A narrative review. J. Neuropsychol. 2025; 19: 26–41. 10.1111/jnp.12390 PMC11923728 39233655

[93] Gil‐Ugidos A , Vázquez‐Millán A , Samartin‐Veiga N , Carrillo‐de‐la‐Peña MT . Conditioned pain modulation (CPM) paradigm type affects its sensitivity as a biomarker of fibromyalgia. Sci. Rep. 2024; 14: 7798. 10.1038/s41598-024-58079-7 PMC10987675 38565572

[94] Marshall A , Rapteas L , Burgess J *et al*. Small fibre pathology, small fibre symptoms and pain in fibromyalgia syndrome. Sci. Rep. 2024; 14: 3947. 10.1038/s41598-024-54365-6 PMC10873371 38365860

[95] Zetterman T , Markkula R , Miettinen T , Kalso E . Heart rate variability responses to cognitive stress in fibromyalgia are characterised by inadequate autonomous system stress responses: A clinical trial. Sci. Rep. 2023; 13: 700. 10.1038/s41598-023-27581-9 PMC9839669 36639565

[96] Grayston R , Czanner G , Elhadd K *et al*. A systematic review and meta‐analysis of the prevalence of small fiber pathology in fibromyalgia: Implications for a new paradigm in fibromyalgia etiopathogenesis. Semin. Arthritis Rheum. 2019; 48: 933–940. 10.1016/j.semarthrit.2018.08.003 30314675

[97] De Peuter S , Diest V I , Vansteenwegen D , Van den Bergh O , JWS V . Understanding fear of pain in chronic pain: Interoceptive fear conditioning as a novel approach. Eur. J. Pain 2011; 15: 889–894. 10.1016/j.ejpain.2011.03.002 21440472

[98] Damasio AR . A second chance for emotion. In: Cognitive Neuroscience of Emotion. Oxford University Press, New York, NY, US, 2000; 12–23 (Series in affective science).

[99] Eccleston C , Crombez G , Aldrich S , Stannard C . Worry and chronic pain patients: A description and analysis of individual differences. Eur. J. Pain 2001; 5: 309–318. 10.1053/eujp.2001.0252 11558986

[100] AJ MD , Rollman GB , GA MC . Generalized hypervigilance in fibromyalgia: Evidence of perceptual amplification. Pain 1996; 66: 133–144. 10.1016/0304-3959(96)03059-x 8880834

[101] Perrot S . Fibromyalgia: A misconnection in a multiconnected world? Eur. J. Pain 2019; 23: 866–873. 10.1002/ejp.1367 30663833

[102] Slater M , Sanchez‐Vives MV . Transcending the self in immersive virtual reality. Computer 2014; 47: 24–30.

[103] Kilteni K , Groten R , Slater M . The sense of embodiment in virtual reality. Presence Teleop. Virt. 2012; 21: 373–387.

[104] Hudson JI , Pope HG . Fibromyalgia and psychopathology: Is fibromyalgia a form of “affective spectrum disorder.”. J. Rheumatol. Suppl. 1989; 19: 15–22. 2691675

[105] Sündermann O , Flink I , Linton SJ . My body is not working right: A cognitive behavioral model of body image and chronic pain. Pain 2020; 161: 1136–1139. 10.1097/j.pain.0000000000001822 32028333

[106] Tsay A , Allen TJ , Proske U , Giummarra MJ . Sensing the body in chronic pain: A review of psychophysical studies implicating altered body representation. Neurosci. Biobehav. Rev. 2015; 52: 221–232. 10.1016/j.neubiorev.2015.03.004 25783221

